# Making Moral Judgments from a World-Historic Standpoint: The Case of Woodrow Wilson

**DOI:** 10.1007/s12115-016-0019-3

**Published:** 2016-03-30

**Authors:** Steve Fuller

**Affiliations:** Department of Sociology, University of Warwick, Coventry, CV4 7AL UK

**Keywords:** Woodrow Wilson, W.E.B. DuBois, *Realpolitik*, World-historic morality, Progressivism, Race, Class

## Abstract

This comment reflects on the recent row over Woodrow Wilson’s legacy, which has focussed on his undeniable racism. This was in pursuit of a ‘Progressive’ agenda which paved the way for the United States to become the geopolitical force that it continues to be today. Without necessarily approving of Wilson’s particular judgements, I argue that he operated with a ‘world-historic’ sense of morality characteristic of *Realpolitik*, a perspective which might be worth considering by those who believe that modern preoccupations with ‘race’, ‘class’ and ‘gender’ will remain part of the sociological firmament indefinitely.ᅟ

One of the great mysteries of American politics is why it was that a former President of Princeton University, a founder of the political science profession in the United States, turns out to be the person who decisively reversed the reforms established in the spirit of the emancipation of Blacks from slavery. The only unequivocally ‘intellectual’ figure to have occupied the Oval Office appears to have been the most overtly racist in his political judgment – barring the nation’s founding fathers, of course.

I speak of Woodrow Wilson, who is known to Europeans as the man who through his brokering of the settlement after the First World War demonstrated wisdom at a global level that had never before been witnessed in Americans. Wilson set the benchmark in international affairs for subsequent American presidents – ‘making the world safe for democracy’ – to which all have aspired but relatively few have met. Moreover, Wilson introduced a variety of domestic measures, not least a national income tax, to redistribute wealth from the rich to the poor. Nevertheless, Wilson permitted – and allowed in the federal government itself – discrimination against Black people.

There is no doubt that Wilson was a ‘racist’ by today’s standards, which is to say, he used racially based arguments publicly to justify his policies. The question is whether this fact tells against him in a way that justifies the removal of his name from buildings, institutes, fellowships, etc. – especially at Princeton, which he converted from a glorified finishing school to a major research-based university. This is the proposal put forward by the Black Justice League, a recent student-based group at Princeton that has made its presence felt to Peter Singer, who nowadays holds an endowed chair in ethics there.

Singer recounts Wilson’s significance much as I have, but then draws this conclusion: “The end result of the conversation we should be having may well be the recognition that to attach Wilson’s name to a college or school sends a message that misrepresents the values for which the institution stands. Yet, presumably ‘the values for which the university stands’ are ones that transcend the decisions made about what is right and wrong at a given time”.

I make this point not in the spirit of excusing Wilson as a victim of his times who failed to see the value of racial justice. After all, Wilson himself had given assurances to America’s leading Black intellectual, W.E.B. DuBois, that he would do justice to Black people – and in return received DuBois’ endorsement in the 1912 presidential election. At the same time, I don’t want to invoke a character flaw on Wilson’s part, which would suggest that somehow racism was so deeply entrenched in his soul as to cloud his judgment. The evidence simply shows that Wilson allowed agencies of the federal government to reintroduce segregation policies if they so wished. These are not the actions of a ‘blind’, let alone a ‘principled’ racist.

I would submit that Wilson knew exactly what he was doing: He believed that whatever evil was committed by allowing segregation at the federal level (and his relative indifference to Black discrimination in the nation at large) was offset by the greater good of national unity that he thought it was serving. To put the matter bluntly: Wilson believed – rightly or wrongly – that racial differences were less significant than class differences in American society, and that the latter had to be addressed even at the expense of the former. In this he was one with fellow ‘Progressive’ politicians and intellectuals of the time, though they handled the matter somewhat differently, depending on the particular circumstances on which they were called to take decisions.

It would be also fair to say – and US Conservative commentators such as Jonah Goldberg have made great sport of this – that the Progressives believed there was a racial dimension to poverty that required both better upbringing and fewer offspring. Moreover, this was in line not only with the emerging ‘Fabian’ movement in the UK that would eventually lead to a split in the Liberal Party which in turn led to the establishment of the Labour Party. It also characterised the considered view of W.E.B. DuBois, who also authored *The Talented Tenth*. All of these people held what nowadays would be branded as ‘eugenicist’ views.

This is not very surprising. Notwithstanding the later Nazi associations, early twentieth century eugenics tended to place considerable ‘Lamarckian’ faith in the value of a good environment in improving the general stock of humanity. (It is worth remarking that recent developments in ‘epigenetics’ may return policymakers to this state of mind in the not-too-distant future.) As it turns out, DuBois’ main grievance with Wilson’s laissez faire attitude towards racial segregation was that it permitted the firing of qualified Black people. So what did Wilson think he was doing?

For Wilson, the first native of the Old Confederacy elected president in the 50 years since the end of the Civil War, the main threat of insurrection in the United States came not from the Blacks – who were fitfully making their way through a reconstructed America – but from the poor Whites who felt they had been given a raw deal in the post-war settlement. To be sure, DuBois had explored this problem in his classic sociological work, *Black Reconstruction in America*, much of which had been published in the years just before Wilson’s election. DuBois believed that poor White hostility to Blacks in the South, which characterized the rise of the Ku Klux Klan and Jim Crow laws, was misplaced class aggression which was better aimed at the rich White landowners who in many cases managed to recover their assets while leaving their former White underlings behind.

From the tenor of DuBois’ writings of the period, it was clear that he would have liked to see the poor Whites and Blacks of the South join together in a great class war against the rich White families, which in turn would have pushed the issue of equality and justice for all to the top of the Washington agenda. Indeed, as the twentieth century wore on, DuBois became increasingly friendly to the politics of revolutionary socialism. He ended a very long life – in 1963 at age 95 -- in a very curious place from a world-historic standpoint: Exiled in Ghana with his US passport revoked, deeply sceptical of Martin Luther King’s non-violent approach to civil rights, and the first American winner of the USSR’s Lenin Peace Prize.

Wilson may well have been familiar with DuBois’ perspective but demurred. What he did instead was to ensure that the South remained firmly within his own Democratic Party for more than 50 years, until racial segregation was finally outlawed in 1964, at which time Southerners began the current trend of voting Republican in presidential elections. What the nation gained from Wilson’s judgment was an electable party dedicated to the interests of working class people capable of leading on the world stage, echoes of which remain in Barack Obama’s presidency. To be sure, the main beneficiary of this judgment was not Wilson himself but his Secretary of the Navy, Franklin Delano Roosevelt.

It is easy to forget that among those who propped up FDR’s New Deal, with its unprecedented level of economic regulation and redistribution, was a reliable phalanx of Southern legislators who in the following generation opposed the Civil Rights Act of 1964. A case in point is J. William Fulbright of Arkansas, after whom the famous international academic fellowships are named and whom Bill Clinton has named as a political mentor. But even FDR himself was only marginally better than Wilson on civil rights matters, understanding that Blacks knew that they would be always more likely to benefit under a Democratic regime – albeit more as poor than as Black people. He proved to be correct in this judgment, however unpalatable it may seem to us today.

I am inclined to believe that Woodrow Wilson’s political judgment was flawed when he made it easier for federal employers to segregate on racial grounds. But this is nothing to do with any ‘racism’ that might be attributed to Wilson. Rather, Wilson may have simply erred in the way he judged claims of class against claims of race. Perhaps he could have ensured largely the same consequences – the ones that history actually bore out from FDR to the Civil Rights Act – without having handled the race issue so clumsily in 1913. Indeed, there might have been some way of avoiding the need for the Civil Rights Act altogether.

To be sure, I may be overestimating the political possibilities available to Wilson. But this is a matter for academic debate, not grounds for stigmatizing someone who, as a matter of fact, did so much to make the United States the positive force in the world that it was in the twentieth century.

Against this backdrop, we should think carefully about the principle on which we dedicate the name of public institutions. If in the past we erred in naming things merely for the positive effects that others have had our own lives, regardless of the effects they had on the people of their day, we should equally avoid the error of removing names without considering what other means was available to bring about the effects for which those named have been celebrated. If there were other means, that case needs to be made explicitly – including the other likely consequences of that counterfactual reality.

Woodrow Wilson was probably ‘racist’ – but in the same sense that Abraham Lincoln or perhaps even FDR was. I refer here to their personal belief in some sense of radical difference – if not inferiority -- of Black vis-à-vis White Americans. However, these politicians acted differently on their shared belief given the different conditions on which they were called to act. Lincoln’s decision to free the slaves was just as politically calculated as Wilson’s to resume segregation. What differed was the context of the decision-making – that’s all, really. I can easily see Lincoln and Wilson each doing what the other did under switched circumstances.

Support for the Black Justice League’s call to remove Wilson’s honoured status at Princeton is most plausible if Wilson’s ‘racism’ is associated with a denial of basic human rights and dignity to Black people. But what if Wilson believed that ‘basic human rights and dignity’ were better addressed not at the level of race but at the level of class? To be sure, the two overlapped significantly in early twentieth century America, insofar as most Blacks were still mired in poverty.

However, in terms of averting a second Civil War from disenfranchised White Southerners, which would impede the power and vision that the United States could bring to the world stage, Wilson may well have calculated that the advancement of Black people could be put on hold. After all, the Blacks were not nearly as well organized in terms of their capacity for violence and disruption as the poor Southern Whites who were flocking to the Ku Klux Kan. DuBois knew this as well, which was an endless source of frustration, even after he founded the National Association for the Advancement of Coloured People in 1909.

Admittedly the above account of Wilson’s reasoning is speculative, but it reflects a man who in line with Progressive thought envisaged politics as both a *big* and a *long* game. Wilson’s Progressive predecessor Theodore Roosevelt had demonstrated America’s adeptness at the big game by brokering the peace in the Russo-Japanese War, for which he won the 1906 Nobel Peace Prize. But it was left to Wilson to demonstrate the nation’s competence at the long game of politics.

The Progressive movement’s debt to the philosophical master of long-term thinking, Hegel, has yet to be fully paid – but Wilson the political science professor obliged as he heaped praise on Otto von Bismarck’s Hegelian synthesis of modern Germany. In particular, he admired Bismarck’s reorganization of the civil service to incorporate potential political opponents and the introduction of a social security system funded by income redistribution. This in turn was widely seen to have averted what Karl Marx had predicted, namely, class revolt by the world’s best organized labour movement.

Wilson himself would probably wish to be judged by Bismarckian *Realpolitik* standards of political ethics. It implies a sense of moral appraisal that is rarely taught in ethics courses. It takes seriously that people can understand themselves in many different ways -- or possess ‘multiple identities’ or embody ‘countervailing interests’. In short, we are always already ‘raced’, ‘classed’, ‘gendered’, ‘speciesed’, etc. But these facts about ourselves matter differently as motivators to action at different times. The ethical challenge then is how to appeal to people to get the best overall result out of them. Or, put the other way round: How do people need to think about themselves to do as much good as possible under the circumstances?

An underlying assumption here is that the person asking these questions knows what would count as a ‘better’ or ‘worse’ result – at least sufficiently well that distinguishing the two states is a meaningful activity. Herein lies the specifically ‘Progressive’ character of moral appraisal, which Bismarckian political ethics presupposes. People are both much more than they have been and can be led to places where they have never been before. Such ideas are commonplaces in utopian discourse but have no obvious place in modern ethics courses, which whatever else they teach tend to proscribe against the ‘instrumentalization’ of the moral agent.

Make no mistake: You cannot aspire to Bismarckian standards of political ethics unless you believe that, in some deep sense, ‘the end justifies the means’. That phrase was originally used in the modern period to characterize the inscrutability of divine justice but was repurposed by Hegel to capture how even the Caesars and Napoleons of this world were ultimately no more than vehicles for the ‘world-historic spirit’, which is on a mission to release humanity’s potential for rational freedom.

Even without arguing the finer points of Hegel’s secular theodicy, it should be clear that different self-understandings of what it means to be human will be relevant to promoting this agenda at different moments in history. Against this backdrop, the Bismarckian looks to which distinctions among humans constitute blockages to further progress. Wilson may have been wrong in privileging class over race so categorically, but if so it was a calculated error which nevertheless served to advance the overall objective. In that case, his was a cognitive not a moral failure, the enormity of which has yet to be agreed.

‘Race’ enjoys a curious status in contemporary folk ontology. It remains something that commands enormous piety in public debates, all the while both biologists and sociologists – in one of their few points of agreement – concur that it is an ambiguous term of reference. Moreover, racial differences that have been politically salient in the past – say, between ‘Blacks’ and ‘Whites’ -- are in the process of being transformed, hybridized and quite possibly eliminated altogether.

A variety of factors are at play. Some, like interracial marriage, are quite obvious in their effects. Others affect our intuitions of racial differences more subtly, such as DNA-based genealogies, which reveal surprising breeding patterns on the part of our ancestors. Indeed, those truly concerned about the long-term relevance of ‘race’ in politics might better spend their energies focusing on the political economy surrounding humanity’s increasing capacity to design its offspring.

No doubt we shall continue to live in a world in which value discriminations among human lives remain rife, and many – if not most – of their grounds will appear prejudiced to those who stand on the wrong side of the divide. However, those grounds change, and those campaigning for social justice should be wary not to act like generals eager to fight the last war, regardless of the present danger. Just as it is easy for generals to treat their memories as a playbook for the current theatre of war, so too humanists with their professional closeness to things already done may too easily suppose that deconstructing a text from the past improves the fate of someone living today.

In terms of the great sociological categories, ‘class’ has for at least for a generation seemed old-fashioned as an effective mode of critique, except in certain nostalgic quarters of Europe’s intelligentsia. This is not to deny the persistence of economically based social inequalities. But a class-based analysis fails to capture either the self-understanding of those subject to the inequalities or any likely policies that might be implemented to address them. ‘Race’ is currently going the same way as class, and ‘gender’ is on the horizon. It may take another century for both politics and science to make a similar point about ‘species’ vivid for most people and salient for public policy. In any case, the ultimate moral lesson is clear: Our categories of moral appraisal are just as vulnerable to error as the judgments of those who we find wanting by them.

